# Social epidemiology of gender diversity in early adolescents

**DOI:** 10.1038/s41390-025-04576-y

**Published:** 2025-11-26

**Authors:** Jason M. Nagata, Karen Li, Shirley S. Sui, Patrick Low, Jonanne Talebloo, Christopher D. Otmar, Iris Yuefan Shao, Kyle T. Ganson, Alexander Testa, Jinbo He, Orsolya Kiss, Claire D. Brindis, Fiona C. Baker

**Affiliations:** 1 Department of Pediatrics, University of California, San Francisco, San Francisco, CA USA; 2 Factor-Inwentash Faculty of Social Work, University of Toronto, Toronto, ON Canada; 3 Department of Management, Policy and Community Health, University of Texas Health Science Center at Houston, Houston, TX USA; 4 School of Humanities and Social Science, The Chinese University of Hong Kong, Shenzhen, Guangdong, China; 5 Center for Health Sciences, SRI International, Menlo Park, CA USA; 6 Philip R. Lee Institute for Health Policy Studies, University of California, San Francisco, San Francisco, CA USA

## Abstract

**Background:**

Few large U.S. cohort studies have examined multidimensional measures of gender diversity; therefore, this study investigates their associations with sociodemographic factors in a national sample of 12- to 13-year-old adolescents.

**Methods:**

We conducted a cross-sectional analysis of Adolescent Brain Cognitive Development (ABCD) Study data (2019–2021, Year 3 follow-up, *N* = 10,089). Associations between sociodemographic characteristics and gender were evaluated using ordinal logistic regression for ordinal measures (gender non-contentedness and gender expression), multinomial regression models for categorical measures (transgender identity and felt gender), and linear regression models for continuous measures (felt gender and gender spectrum).

**Results:**

Gender spectrum measures revealed the most diverse responses: 25.9% of sex-assigned females self-describing masculinity and 9.9% of sex-assigned males self-describing femininity. Female sex assigned at birth was associated with greater gender-diverse responses across all continuous and categorical gender measures compared to male sex assigned at birth. Higher household income was associated with less gender diversity relative to lower household income.

**Conclusion:**

Assessment of gender across multiple measures beyond binary gender identity (e.g., transgender vs. cisgender) may yield more diversity of responses in early adolescents. Future research could explore explanations for the sociodemographic factors associated with greater gender diversity in early adolescents.

**Impact:**

- Our study uses a large and diverse cohort to analyze sociodemographic associations with gender diversity using multidimensional measures.
- Across gender measures, female sex assigned at birth and lower household income were associated with greater gender diversity.
- Future research on gender diversity in early adolescents should use multidimensional gender measures to better understand the nuances of gender development.

## Introduction

Gender is a multidimensional facet of an individual’s identity, encompassing attitudes, feelings, and behaviors that a culture associates with a person’s sex assigned at birth.^1^ A significant portion of gender formation occurs during adolescence when individuals explore their gender through both biological and social processes.^2^ Gender consists of various dimensions, each with distinct developmental patterns influenced by hormonal signaling and time spent in gendered social contexts.^3^ During this period, many adolescents may experience conflict between their gender identity and sex assigned at birth, leading to gender non-contentedness, defined by low satisfaction with one’s gender.^4^

One area of concern is the poor health outcomes linked to diverse gender identities. For example, gender minorities, or individuals whose gender identity differs from their sex assigned at birth, experience disproportionately higher rates of physical and mental health morbidities compared to cisgender individuals.^5^ Additionally, gender non-conforming and transgender adolescents face significant mental health challenges that may extend into adulthood. These include high rates of depressive symptoms and suicidal ideation, with one study finding 37.4% of participants endorsing some presence of suicidal ideation.^6–9^ These findings highlight a substantial gap in health outcomes between gender minority and cisgender adolescents, as well as the importance of studying the roots of these differences in health outcomes even among younger adolescents in the U.S.

Gender has traditionally been viewed as a binary dichotomization between cisgender and transgender. Currently, the majority of investigations assessing gender use this binary definition of gender in their methodology.^10^ However, gender has been increasingly recognized as a multifaceted concept that may be more accurately described through different dimensions along a continuous spectrum.^11^ For example, diverse gender does not necessitate transgender identity, and many adolescents experiencing discomfort with their gender or curiosity about their gender expression may not claim an identity that differs from their sex assigned at birth.^12^ Using multifaceted definitions of gender, which are more nuanced and accurate in describing diverse aspects of gender, is important in describing adolescents who think broadly about their gender.

With increasing recognition of the diversity of gender, there is a need for data elucidating sociodemographic associations with gender to provide targeted support for gender minority early adolescents.^13^ Sociodemographic factors may play an important role in shaping one’s gender as well as one’s relationship with their identity. Factors associated with reporting gender diversity and transgender identity include lower maternal education, increased traumatic family life events, lack of family cohesion, perceived lack of family economic resources, and female sex.^14^ Moreover, there is currently sparse literature describing gender in early adolescence, which is an important period in the development of gender identity and dysphoria.^15^ Individual differences in the age when physical changes emerge vary widely, with a typical onset between 8 and 13 years in females and 9 and 14 years in males.^16^ Although several studies examine sociodemographic factors associated with gender in adolescents, there is a paucity of literature assessing these associations during early adolescence and specifically measuring gender diversity using multidimensional measures of gender that capture nuances of an individual’s unique gender experience. To address these gaps, our study aims to assess sociodemographic associations of gender diversity using multidimensional measures of gender in a demographically diverse national sample of early adolescents in the U.S.

## Methods

### Study population

The Adolescent Brain Cognitive Development (ABCD) Study is a longitudinal cohort study tracking the health and cognitive development of 11,875 adolescents from 21 research sites across the United States (baseline 2016–2018). Informed consent was obtained from caregivers, and assent was secured from each participating adolescent. The University of California, San Diego institutional review board (IRB) approved the study, and local IRB approval was granted at each participating site. Further details on the structure of the study and recruitment of participants are outlined elsewhere.^17^ The current study utilizes data that included gender identity and sex at birth information from the Year 3 follow-up (2019–2021), resulting in an analytic sample of 10,089 adolescents (Appendix A).

### Measures

#### Sociodemographic characteristics

Sex at birth was categorized as male or female. Race/ethnicity was defined as a categorical variable with options being White, Latino, Black, Asian, Native American, and Other. Parent education was a binary variable defined as “high school or less” and “more than high school”. Household income was defined as a six-level categorical variable ranging from the lowest yearly income category of less than $25,000 to the highest of over $200,000 per year. These variables were based on parents’ self-reports and were collected at baseline. The research site was also included as a covariate to adjust for potential regional variation.

#### Gender

Using data from the ABCD Youth Gender Survey,^18^ we assessed gender through two continuous measures (felt gender and self-described placement on a gender spectrum), two ordinal measures (gender non-contentedness and gender expression), and two categorical measures (felt gender and transgender identity). All measures were coded such that higher scores indicated self-identification of being gender diverse. Complete measures, including item wording, are included in Table 1, and definitions of gender constructs are included in Appendix C.

**Table 1:** Sociodemographic characteristics & self-reported gender responses for early adolescents (age range: 12–13 years) at the Year 3 follow-up of the Adolescent Brain Cognitive Development (ABCD) Study, 2019-2021 (*N* = 10,089)

Response	Mean (SD) / %	
**Sociodemographic characteristics**		
Age at Year 3 (years)	12.91 (0.97)	
Sex (%)		
Male	51.7%	
Female	48.3%	
Race/ethnicity (%)		
White	54.4%	
Latino	19.9%	
Black	15.6%	
Asian	5.5%	
Native American	3.1%	
Other	1.4%	
Household income (%)		
Less than $25,000	16.3%	
$25,000 to <$50,000	20.0%	
$50,000 to <$75,000	17.9%	
$75,000 to <$100,000	14.0%	
$100,000 to <$200,000	24.2%	
$200,000 or higher	7.7%	
Parents’ highest education (%)		
More than high school	82.1%	
High school or less	17.9%	
**Self-reported gender identity responses**		
Are you transgender?		
Yes	1.0%	
Maybe	1.1%	
No	97.9%	
Has this caused any problems for you with your family or with kids at school?		
Not at all	60.1%	
Some	28.8%	
A lot	11.1%	
What was your sex assigned at birth, on your birth certificate?		
Male	51.6%	
Female	48.4%	
What is your current gender identity?		
Boy (male at birth)	51.4%	
Girl (male at birth)	0.2%	
Another gender (e.g. nonbinary) (male at birth)	0.2%	
Girl (female at birth)	45.7%	
Boy (female at birth)	0.6%	
Another gender (e.g. nonbinary) (female at birth)	1.9%	
**Felt gender group**^**a**^		
Cisgender/sex congruent felt gender (SCFG)	79.6%	
1-STEP	8.3%	
2-STEP	5.0%	
Minority Group	7.0%	
**Self-reported gender responses (Youth Gender Survey)**	Male at birth	Female at birth
How much do you feel like a …	… boy?	… girl?
Not at all	0.6%	1.7%
A little	0.5%	2.5%
Somewhat	1.2%	5.8%
Mostly	6.0%	17.2%
Totally	91.8%	72.9%
How much do you feel like a …	girl?	boy?
Totally	0.3%	0.6%
Mostly	0.3%	2.3%
Somewhat	0.7%	5.4%
A little	3.4%	15.5%
Not at all	95.3%	76.3%
How much have you had the wish to be a …	… girl?	… boy?
Always	0.2%	1.1%
Often	0.3%	2.3%
Sometimes	0.9%	5.8%
Rarely	5.9%	15.3%
Never	92.7%	75.4%
How much have you dressed or acted as a …	… girl during play?	… boy during play?
Always	0.1%	1.4%
Often	0.4%	4.4%
Sometimes	1.6%	11.3%
Rarely	8.0%	20.0%
Never	90%	62.9%
How do you think other people would describe you?^2^		
Very feminine	0.4%	28.6%
Mostly feminine	0.4%	31.9%
Somewhat feminine	2.2%	15.6%
Equally feminine and masculine	8.6%	18.1%
Somewhat masculine	16.2%	4.2%
Mostly masculine	31.3%	1.4%
Very masculine	40.9%	0.3%
How would you describe yourself?^b^		
Very feminine	0.4%	29.9%
Mostly feminine	0.5%	30.9%
Somewhat feminine	1.2%	13.3%
Equally feminine and masculine	7.8%	20.5%
Somewhat masculine	14.5%	3.1%
Mostly masculine	27.1%	1.8%
Very masculine	48.5%	0.5%

ABCD Study propensity weights were applied based on the American Community Survey from the U.S. Census.

^**a**^As defined in Potter et al., 2021; SCFG = sex congruent felt gender

^b^These questions were introduced to the ABCD Youth Gender Survey part way through the 3-year follow-up, and as such had a high degree of missing values (54.8% overall).

#### Felt gender

Felt gender was analyzed as a categorical variable using the felt gender group structure presented in Potter et al. (2021) and as a continuous variable (Appendix C).^18^ For felt gender groups, adolescents were categorized into four groups (cisgender/sex-congruent felt gender, 1-STEP, 2-STEP, minority group) based on their responses to the survey questions “How much do you feel like a boy?” and “How much do you feel like a girl?” For the continuous variable, an average summary score was generated by taking the mean score across the two questions for each respective sex and reverse-coded such that a higher score represents higher gender diversity, ranging from 1 (most gender conforming) to 5 (most gender diverse).

#### Gender non-contentedness

Gender non-contentedness was measured as an ordinal measure based on the responses to the question “How much have you had the wish to be a girl?” for adolescents assigned male at birth. The question “How much have you had the wish to be a boy?” was asked to adolescents assigned female at birth. Responses were given on a 5-item Likert-type scale from 1 (*Always*) to 5 (*Never*) and reverse-coded such that higher scores indicate higher gender diversity.

#### Gender expression

Gender expression was analyzed as an ordinal variable. Participants assigned male at birth were asked, “How much have you dressed or acted as a girl during play?”, and responses were recorded on a 5-item Likert scale from 1 *(Always)* to 5 *(Never)*. The same scale was used for participants assigned female sex at birth. Responses were reverse-coded such that higher scores indicate higher gender diversity.

#### Gender spectrum

The gender spectrum questions “How would you describe yourself?” and “How do you think others would describe you?” were added partway through the Year 3 follow-up (see Appendix B for sociodemographic comparisons of those with versus without missing gender spectrum data). Responses to both questions were separately recorded using a 7-point Likert scale from 1 (*Very masculine* for male at birth, *Very feminine* for female at birth) to 7 (*Very feminine* for male at birth, V*ery masculine* for female at birth). To create the gender spectrum measure as a continuous variable, these responses were directly averaged such that a higher score indicates higher gender diversity.

#### Transgender identity

Transgender identity was analyzed as a categorical variable based on the question “Are you transgender?” Responses included yes, maybe, no, I do not understand, and refuse to answer.

### Statistical analyses

Statistical analyses were performed using R version 4.4.0. Descriptive statistics, including percentages, means, medians, and standard deviations were calculated. Linear regressions were used to evaluate the associations between sociodemographic characteristics and two continuous measures of gender (felt gender and self-described placement on a gender spectrum). Ordinal logistic regressions were used to evaluate the associations between sociodemographic characteristics and two ordinal measures of gender (gender non-contentedness and gender expression). Finally, multinomial regressions were used to evaluate the associations between sociodemographic characteristics and two categorical measures of gender (transgender identity and felt gender). Linear and ordinal models were fit using the *survey* package version 4.4-2, while multinomial models were fit using the *svyVGAM* package version 1.2. Each model included age, sex assigned at birth, race/ethnicity, parental education, and household income as key exposures, with research site as a covariate. Additionally, to align our sample with demographic profiles represented in the American Community Survey and enhance the representativeness of our findings, all descriptive statistics and regression models incorporated the ABCD propensity weights.^19^

## Results

In our sample of 10,089 eligible adolescents (M_age_=12.9, SD_age_ = 0.97), 48.3% were assigned female sex at birth and 45.6% were from racial/ethnic minority groups. Gender-diverse responses were uncommon for all gender questions examined in this study, but they occurred more frequently for those assigned female at birth (Table 1).

We found several significant associations between various sociodemographic characteristics and the various gender diversity variables (Tables 2–4). Female sex assigned at birth had significant associations with greater gender diversity across all gender variables. For instance, female sex at birth was associated with greater odds of reporting gender non-contentedness (OR 4.34, 95% CI 3.74–5.03), diverse gender expression (OR 5.52, 95% CI 4.87–6.27), and identifying as transgender vs. not identifying as transgender (OR 9.11, 95% CI 4.56–18.21) (Tables 2–4). Higher household income categories were generally associated with less gender diversity compared to the lowest income category (<$25,000) across various gender diversity variables, except for gender identity.

**Table 2:** Associations between sociodemographic characteristics and ordinal/continuous measures of gender diversity in Year 3 of the Adolescent Brain Cognitive Development (ABCD) Study

Sociodemographic characteristics	Felt gender		Gender non-contentedness	Gender expression	Gender spectrum	
	*B* (95% CI)	*p*	*OR (95% CI)*	*OR (95% CI)*	*B* (95% CI)	*p*
Age	−0.019 (−0.041, 0.003)	0.091	0.98 (0.88, 1.09)	1.03 (0.96, 1.11)	−0.012 (−0.061, 0.036)	0.616
Sex						
Male (reference)						
Female	**0.30 (0.27, 0.32)**	**<0.001**	**4.34 (3.74, 5.03)**	**5.52 (4.87, 6.27)**	**0.498 (0.419, 0.578)**	**<0.001**
Race/ethnicity						
White (reference)						
Latino	−0.019 (−0.071, 0.033)	0.484	0.97 (0.76, 1.23)	**0.69 (0.55, 0.86)**	0.000 (−0.139, 0.138)	0.996
Black	−0.005 (−0.052, 0.041)	0.817	0.88 (0.71, 1.09)	0.90 (0.74, 1.09)	−0.053 (−0.179, 0.073)	0.41
Asian	−**0.058 (−0.109, −0.007)**	**0.027**	1.04 (0.77, 1.41)	0.75 (0.56, 1.00)	0.062 (−0.126, 0.250)	0.517
Native American	0.007 (−0.076, 0.089)	0.871	1.01 (0.67, 1.53)	1.11 (0.76, 1.62)	0.246 (−0.025, 0.517)	0.075
Other	−0.079 (−0.194, 0.035)	0.174	0.61 (0.28, 1.33)	0.96 (0.52, 1.76)	−0.102 (−0.600, 0.396)	0.689
Household income (%)						
Less than $25,000 (reference)						
$25,000 to <$50,000	−0.023 (−0.081, 0.036)	0.446	**0.75 (0.59, 0.95)**	**0.72 (0.57, 0.90)**	−0.043 (−0.195, 0.110)	0.584
$50,000 to <$75,000	−**0.066 (−0.126, −0.006)**	**0.031**	**0.68 (0.52, 0.88)**	**0.70 (0.55, 0.90)**	−0.022 (−0.184, 0.141)	0.793
$75,000 to <$100,000	−**0.093 (−0.153, −0.034)**	**0.002**	**0.55 (0.42, 0.72)**	**0.71 (0.55, 0.90)**	−**0.184 (−0.347, −0.021)**	**0.027**
$100,000 to <$200,000	−**0.102 (−0.160, −0.044)**	**0.001**	**0.51 (0.40, 0.66)**	**0.64 (0.51, 0.81)**	−**0.345 (−0.497, −0.192)**	**<0.001**
$200,000 or higher	−**0.132 (−0.195, −0.069)**	**<0.001**	**0.39 (0.29, 0.53)**	**0.59 (0.45, 0.78)**	**−0.403 (−0.575, −0.231)**	**<0.001**
Parents’ highest education						
More than high school (reference)						
High school or less	0.027 (**−**0.024, 0.079)	0.299	**0.77 (0.61, 0.97)**	0.84 (0.68, 1.03)	**−**0.09 (**−**0.222, 0.041)	0.179

Bold indicates *p *< 0.05; B > 0 and OR > 1 are more gender diverse. Felt gender and gender spectrum were run as linear regressions. Gender non-contentedness and gender expression were run as ordinal logistic regressions. All models include age, sex, race/ethnicity, household income, parent education, and site. ABCD Study propensity weights were applied based on the American Community Survey from the U.S. Census.

**Table 3:** Associations between sociodemographic characteristics and felt gender groups in Year 3 of the Adolescent Brain Cognitive Development (ABCD) Study

Sociodemographic characteristics	1-STEP: Cisgender	2-STEP: Cisgender	Minority group: Cisgender
	OR (95% CI)	OR (95% CI)	OR (95% CI)
Age	0.89 (0.78, 1.00)	0.99 (0.87, 1.13)	1.00 (0.88, 1.13)
Sex			
Male (reference)			
Female	**6.45 (5.07, 8.20)**	**2.02 (1.51, 2.71)**	1.06 (0.74, 1.52)
Race/ethnicity			
White (reference)			
Latino	1.11 (0.77, 1.59)	**1.63 (1.05, 2.55)**	**2.01 (1.18, 3.42)**
Black	1.14 (0.85, 1.52)	1.29 (0.88, 1.87)	1.47 (0.95, 2.28)
Asian	0.56 (0.30, 1.05)	0.50 (0.25, 1.00)	0.60 (0.27, 1.34)
Native American	1.10 (0.61, 2.00)	1.22 (0.55, 2.70)	0.84 (0.38, 1.86)
Other	0.90 (0.33, 2.46)	1.43 (0.36, 5.73)	1.52 (0.31, 7.48)
Household income (%)			
Less than $25,000 (reference)			
$25,000 to <$50,000	0.84 (0.62, 1.15)	0.92 (0.61, 1.38)	0.79 (0.49, 1.26)
$50,000 to <$75,000	**0.64 (0.45, 0.91)**	0.83 (0.52, 1.31)	0.89 (0.52, 1.51)
$75,000 to <$100,000	**0.52 (0.36, 0.76)**	0.67 (0.42, 1.08)	0.72 (0.42, 1.25)
$100,000 to <$200,000	**0.49 (0.35, 0.69)**	0.82 (0.53, 1.28)	0.73 (0.44, 1.21)
$200,000 or higher	**0.38 (0.24, 0.59)**	0.58 (0.33, 1.01)	0.91 (0.48, 1.73)
Parents’ highest education			
More than high school (reference)			
High school or less	0.97 (0.73, 1.30)	0.78 (0.55, 1.13)	0.91 (0.59, 1.40)

ABCD Study propensity weights were applied based on the American Community Survey from the U.S. Census.

All models include age, sex, race/ethnicity, household income, parent education, and site.

Bold indicates *p* < 0.05; OR > 1 indicates higher odds of gender diversity.

**Table 4:** Associations between sociodemographic characteristics and transgender identity (“Are you transgender?”) in Year 3 of the Adolescent Brain Cognitive Development (ABCD) study

Sociodemographic characteristics	Yes:No	Maybe:No
	*OR (95% CI)*	*OR (95% CI)*
Age	1.23 (0.87, 1.75)	1.20 (0.90, 1.61)
Sex		
Male (reference)		
Female	**9.11 (4.56, 18.21)**	**3.75 (2.14, 6.57)**
Race/ethnicity		
White (reference)		
Latino	1.09 (0.38, 3.11)	**0.25 (0.09, 0.71)**
Black	0.64 (0.31, 1.32)	0.48 (0.23, 1.03)
Asian	0.48 (0.17, 1.35)	0.78 (0.24, 2.47)
Native American	(-)	(-)
Other	(-)	(-)
Household income (%)		
Less than $25,000 (reference)		
$25,000 to <$50,000	0.90 (0.37, 2.18)	1.26 (0.51, 3.11)
$50,000 to <$75,000	0.69 (0.25, 1.88)	1.36 (0.53, 3.51)
$75,000 to <$100,000	**0.29 (0.09, 0.94)**	1.00 (0.39, 2.59)
$100,000 to <$200,000	0.69 (0.27, 1.79)	0.70 (0.28, 1.73)
$200,000 or higher	0.61 (0.21, 1.77)	1.50 (0.55, 4.03)
Parents’ highest education		
More than high school (reference)		
High school or less	0.98 (0.40, 2.38)	0.55 (0.21, 1.46)

ABCD Study propensity weights were applied based on the American Community Survey from the U.S. Census.

All models include age, sex, race/ethnicity, household income, parent education, and site.

Bold indicates *p* < 0.05; OR > 1 indicates higher odds of gender diversity.

(-) indicates insufficient data

We found mixed results for age, race, and parental education, though some patterns did emerge within select racial/ethnic minority groups (Tables 2–4). For instance, Asian race was associated with lower felt gender score compared to White race (B −0.06, 95% CI **−**0.11 to −0.01). Latino adolescents had more diverse felt gender categories compared to White adolescents (OR 2.01, 95% CI 1.18–3.42 for the minority versus cisgender groups; OR 1.63, 95% CI 1.05–2.55 for the 2-STEP vs cisgender groups). In contrast, Latino adolescents reported lower diversity in gender expression compared to White adolescents (OR 0.69, 95% CI 0.55–0.86).

## Discussion

In this demographically diverse sample of early adolescents aged 12–13 years, we found noteworthy sociodemographic associations with multiple measures of gender. For example, female sex assigned at birth was significantly associated with higher odds of gender-diverse responses. Adolescents from households with higher incomes had lower odds of gender-diverse responses relative to adolescents from households with lower incomes. Our observations suggest that gender reporting in early adolescents exhibits differences related to how gender is assessed through sociodemographic characteristics.

In terms of specific sociodemographic characteristics, we found sex differences in gender, with early adolescents who were born female having higher odds of reporting diverse gender responses compared to their male counterparts. This finding is in line with previous research demonstrating higher rates of gender-nonconforming identities in female adolescents relative to male adolescents.^20^ In particular, Potter et al. similarly reported that female participants showed greater diversity in gender expression compared to males in a prior ABCD study analyzing mental health outcomes in Year 1 follow-up.^21^ Given the results of our study, higher gender diversity among female adolescents may be consistent across the cohort, an association that could potentially be explained by higher social tolerance for gender nonconformity among female adolescents.^22^ Heightened sensitivity to emotional stimuli and increased physical body changes during puberty may also contribute to this association, and future studies could investigate the relationship between puberty timing and sex in relation to gender.^3,23^

Our study also showed differences in gender based on household income, with adolescents from higher-income households having lower odds of reporting gender-diverse responses relative to adolescents from lower-income households. This finding aligns with previous research suggesting that socioeconomic status can influence the expression of gender. For example, a previous investigation conducted by Kaltiala et al. found that adolescents from families with lower economic resources were more likely to report transgender identities, which may be due to the higher prevalence of stressful life events in households with lower income playing a role in shaping developmental pathways of adolescent gender.^14,24^ Additionally, it has been shown that individuals from higher social classes may attach more importance to identities indicative of their status,^25^ and adolescents in higher-income families may experience environments with stricter adherence to conventional gender roles. Another possible explanation for this association is selection bias, given that parental education did not show the same effect. Future research is needed to explore mediating factors in the relationship between household income and gender.

Regarding the effect of age, race, and parental education on gender in adolescents, we found mixed results. However, we did note some interesting patterns for certain racial/ethnic minority groups. For instance, adolescents who were reported as being Asian had higher odds of gender-conforming responses in the continuous felt gender measure, with non-significant but consistent associations found in several other measures. Latino adolescents had higher odds of gender diversity in the categorical felt gender group (2-STEP and Minority Group), but lower odds of gender diversity in the ordinal gender expression measure. This may be explained by the prevalence of stigma in the setting of cultural factors such as machismo and marianismo embedded within Latinx communities.^26^ These results are inconclusive but suggest a potential role for cultural and social norms and expectations in gender development within racial minority groups that warrants further research.

Notable strengths of this study include our large, national, diverse sample of early adolescents in the United States and the opportunity for adolescent self-reports using nuanced exposure and outcome measures. Additionally, in conducting this investigation, we used multidimensional measures of gender, including felt gender, gender non-contentedness, gender expression, and gender spectrum. Previous literature has examined the utility of binary dichotomization of gender in survey questionnaires, which raised concern for false positive responses from gender non-minority respondents.^27^ Our investigation used summary scores to analyze gender on a continuous scale, which more accurately captures variations in individual experiences of gender.^11^ Future investigations should continue to include the use of multidimensional and continuous measurements of gender across the life course of early-middle-and late adolescence, as well as young adulthood.

Our investigation has several limitations. First, this study is cross-sectional, and thus, causality cannot be determined. Additionally, data on self-identified placement on a gender spectrum were limited compared to other measures because the questions were added partway through Year 3, and there were some significant differences between adolescents with and without this data. Given the sensitivity of asked questions and the self-reported nature of the survey, there is also a possibility of reporting bias in instances where adolescents do not answer questions truthfully either intentionally or because they do not fully understand the question. Specifically, one question assessing transgender identification may be especially difficult for younger adolescents to interpret and may result in inaccurate responses,^18^ which has been observed in both the current study and other investigations assessing sexual and gender minority identity.^28^

## Conclusion

Our study highlights the complexity of gender within early adolescents, especially the relationship between gender and sociodemographic characteristics, with higher odds of gender diversity associated with being assigned female at birth and coming from lower-income households. These findings demonstrate the importance of considering these factors in understanding adolescent gender development. Future studies should continue using multidimensional, continuous measurements of gender to explore mechanisms influencing these associations. Additionally, diverse samples of youth should also be recruited to investigate the role of cultural context in youths’ experiences of gender identity. The use of multidimensional measurements can strengthen future research and public health efforts to support the needs of gender-diverse early adolescents.

## Supplementary information


Supporting Information


## Data Availability

Data used in the preparation of this article were obtained from the ABCD Study (https://abcdstudy.org), held in the NIH Brain Development Cohorts (NBDC) Portal. J.M.N. had full access to the data in the study and takes responsibility for the integrity of the data and the accuracy of the data analysis.

## Additional Information

The ABCD Study was supported by the National Institutes of Health and additional federal partners under award numbers U01DA041022, U01DA041025, U01DA041028, U01DA041048, U01DA041089, U01DA041093, U01DA041106, U01DA041117, U01DA041120, U01DA041134, U01DA041148, U01DA041156, U01DA041174, U24DA041123, and U24DA041147. A full list of supporters is available at https://abcdstudy.org/federal-partners/. A listing of participating sites and a complete listing of the study investigators can be found at https://abcdstudy.org/principal-investigators.html. ABCD consortium investigators designed and implemented the study and/or provided data but did not necessarily participate in the analysis or writing of this report.
