# Supplementary material for: Social epidemiology of gender diversity in early adolescents
Source: Pediatr Res. 2025 Nov 26;100(1):168–75. doi: 10.1038/s41390-025-04576-y (PMC13524781; doi:10.1038/s41390-025-04576-y)
Supplement: Supplementary file 1 [file 41390_2025_4576_MOESM1_ESM.pdf]

Appendix A. Sociodemographic characteristics of adolescents included vs. excluded in the analysis.

Sociodemographic characteristics	Excluded (N=1771)	Included (N=10089)	p-value
Age (years)	11.7 (3.98)	12.9 (0.942)	
Missing	1525 (86.1%)	0 (0%)	<0.001
Sex (%)			
Male	878 (49.6%)	5303 (52.6%)	0.0344
Female	885 (50.0%)	4786 (47.4%)	
Missing	8 (0.5%)	0 (0%)	
Race/ethnicity (%)			
White	682 (38.5%)	5480 (54.3%)	<0.001
Latino	336 (19.0%)	1695 (16.8%)	
Black	560 (31.6%)	1832 (18.2%)	
Asian	93 (5.3%)	616 (6.1%)	
Native American	64 (3.6%)	346 (3.4%)	
Other	18 (1.0%)	91 (0.9%)	
Missing	18 (1.0%)	29 (0.3%)	
Household income (%)			
Less than $25,000	431 (24.3%)	1200 (11.9%)	<0.001
$25,000 to <$50,000	271 (15.3%)	1315 (13.0%)	
$50,000 to <$75,000	202 (11.4%)	1296 (12.8%)	
$75,000 to <$100,000	163 (9.2%)	1406 (13.9%)	
$100,000 to <$200,000	311 (17.6%)	2999 (29.7%)	
$200,000 or higher	139 (7.8%)	1109 (11.0%)	
Missing	254 (14.3%)	764 (7.6%)	
Parents’ highest education (%)			
More than high school	1240 (70.0%)	8559 (84.8%)	<0.001
High school or less	520 (29.4%)	1519 (15.1%)	
Missing	11 (0.6%)	11 (0.1%)	

Appendix B. Sociodemographic characteristics of adolescents not missing vs. missing gender spectrum data in Year 3 of the ABCD study (2019-2021).

Sociodemographic characteristics	Missing (N=5533)	Not missing (N=4556)	p-value
Age (years)	12.9 (0.830)	12.9 (1.06)	
Sex (%)			0.944
Male	2906 (52.5%)	2397 (52.6%)	
Female	2627 (47.5%)	2159 (47.4%)	
Race/ethnicity (%)			<0.001
White	3239 (58.5%)	2241 (49.2%)	
Latino	902 (16.3%)	793 (17.4%)	
Black	812 (14.7%)	1020 (22.4%)	
Asian	327 (5.9%)	289 (6.3%)	
Native American	193 (3.5%)	153 (3.4%)	
Other	47 (0.8%)	44 (1.0%)	
Missing	13 (0.2%)	16 (0.4%)	
Household income (%)			<0.001
<$25,000	569 (10.3%)	631 (13.8%)	
$25,000-<$50,000	722 (13.0%)	593 (13.0%)	
$50,000-<$75,000	721 (13.0%)	575 (12.6%)	
$75,000-<$100,000	811 (14.7%)	595 (13.1%)	
$100,000-<$200,000	1685 (30.5%)	1314 (28.8%)	
More than $200,000	651 (11.8%)	458 (10.1%)	
Missing	374 (6.8%)	390 (8.6%)	
Parents' highest education (%)			<0.001
More than high school	4796 (86.7%)	3763 (82.6%)	
High school or less	730 (13.2%)	789 (17.3%)	
Missing	7 (0.1%)	4 (0.1%)	

Appendix C. Gender terminology and definitions from the ABCD study

Term	Definition	Question in ABCD
Felt Gender (continuous)	The degree to which an adolescent experiences congruence between their subjective feeling like a boy or girl and their assigned sex at birth (Potter et al. 2021).	How much do you feel like a boy/girl?
		How much do you feel like a girl/boy?
Felt Gender Group	The degree to which an adolescent experiences congruence between their subjective feeling like a boy or girl and their assigned sex at birth (Potter et al. 2021). Adolescents' responses to the survey questions were used to categorize participants as cisgender (0), 1-STEP (1), 2-STEP (2), or Minority Group (3). For participants whose sex assigned at birth is male, survey responses were recorded on a 5-point Likert-type scale from 1 (Not at all) to 5 (Totally) for "How much do you feel like a boy?" and 1 (Totally) to 5 (Not at all) for "How much do you feel like a girl?". The same scale was applied for participants whose sex assigned at birth is female. Responses were coded such that if participants assigned male at birth felt entirely like a boy (5) and not at all like a girl (5) they were categorized as cisgender. If participants assigned male at birth felt totally like a boy (5) but a little like a girl (4) or mostly like a boy (4) and not at all like a girl (5) they were categorized as 1-STEP. If participants assigned male at birth felt mostly like a boy (4) and a little like a girl (4) they were coded as 2-STEP. Finally, any other combination of responses were categorized as Minority Group.	How much do you feel like a boy/girl?
		How much do you feel like a girl/boy?
Gender Contentedness	Happiness with being the gender aligned with sex. Gender non-contentedness is assessed in this study. (Potter et al. 2021).	How much have you had the wish to be a girl/boy?
Gender Expression	The communication of gender through appearance, mannerisms, etc. (Potter et al. 2021).	How much have you dressed or acted as a girl/boy during play?
Gender Spectrum	Concept that recognizes gender as a continuum that encompasses a wide range of gender identities and expressions beyond the traditional binary understanding of male and female, including identities that are very masculine, very feminine, and anywhere in between, as well as identities that may not fit within the traditional definitions of masculinity and femininity at all, such as non-binary and gender-fluid (Hyde et al. 2019, Gulgoz et al. 2022, Richards et al. 2016).	How do you think other people would describe you?
		How would you describe yourself?
Transgender	An umbrella term encompassing those whose gender identities or gender roles differ from those typically associated with the sex they were assigned at birth (APA 2018).	Are you transgender?
